# Supplementary material for: The interactome of intact mitochondria by cross-linking mass spectrometry provides evidence for coexisting respiratory supercomplexes
Source: Mol Cell Proteomics. 2017 Dec 8;17(2):216–32. doi: 10.1074/mcp.RA117.000470 (PMC5795388; doi:10.1074/mcp.RA117.000470)
Supplement: Supplemental Data [file supp_RA117.000470_133922_0_supp_23978_fzffwf.zip › spectra_annotation/mito_DR_spectra_annotation/436-1-12-1-5-1.pdf]

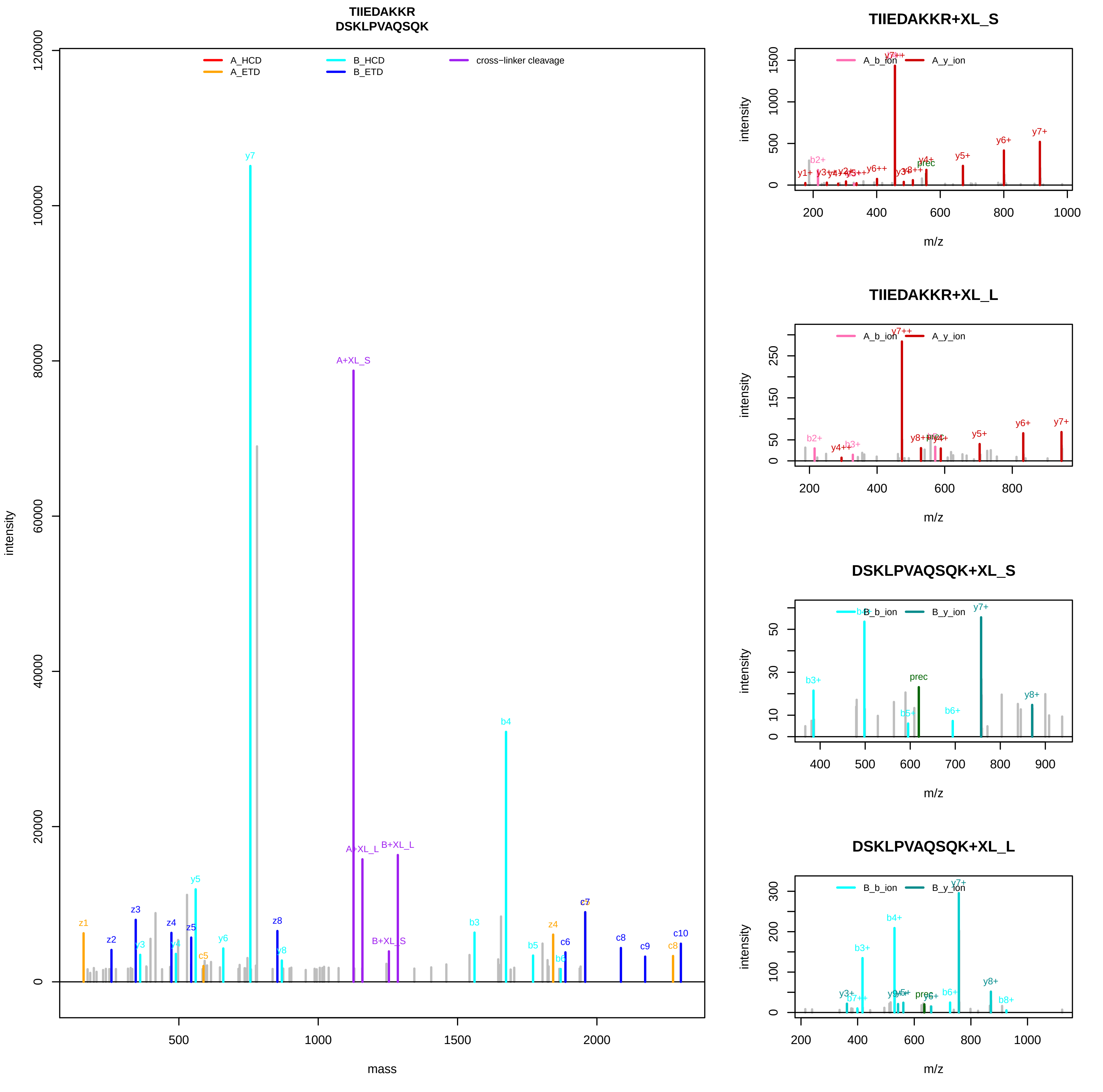

TIIEDAKKR
DSKLPVAQSQK
A_HCD
A_ETD
B_HCD
B_ETD
cross−linker cleavage
intensity
mass
0
20000
40000
60000
80000
100000
120000
500
1000
1500
2000
y7
A+XL_S
A+XL_L
B+XL_L
B+XL_S
b4
y5
z1
z2
z3
y3
z4
y4
z5
c5
y6
z8
y8
b3
b5
z4
b6
c6
c7
c8
c9
c8
c10
TIIEDAKKR+XL_S
TIIEDAKKR+XL_L
DSKLPVAQSQK+XL_S
DSKLPVAQSQK+XL_L
A_b_ion
A_y_ion
B_b_ion
B_y_ion
m/z
intensity
y7++
y7+
y6+
y5+
y4+
prec
y6++
b2+
y1+
y4++
b3+
y8++
b3+
b4+
b5+
b6+
y7+
y8+
b7++
b8+
